# Effect of Boron Addition on the Thermal, Degradation, and Cytocompatibility Properties of Phosphate-Based Glasses

**DOI:** 10.1155/2013/902427

**Published:** 2013-08-07

**Authors:** Nusrat Sharmin, Muhammad S. Hasan, Andrew J. Parsons, David Furniss, Colin A. Scotchford, Ifty Ahmed, Chris D. Rudd

**Affiliations:** ^1^Division of Materials, Mechanics and Structures, Faculty of Engineering, University of Nottingham, Nottingham NG7 2RD, UK; ^2^Division of Electrical Systems and Optics, Faculty of Engineering, University of Nottingham, Nottingham NG7 2RD, UK

## Abstract

In this study eight different phosphate-based glass compositions were prepared by melt-quenching: four in the (P_2_O_5_)_45_-(CaO)_16_-(Na_2_O)_15-*x*_ -(MgO)_24_-(B_2_O_3_)_*x*_ system and four in the system (P_2_O_5_)_50_-(CaO)_16_-(Na_2_O)_10-*x*_-(MgO)_24_-(B_2_O_3_)_*x*_, where *x* = 0,1, 5 and 10 mol%. The effect of B_2_O_3_ addition on the thermal properties, density, molar volume, dissolution rates, and cytocompatibility were studied for both glass systems. Addition of B_2_O_3_ increased the glass transition (*T*_g_), crystallisation (*T*_c_), melting (*T*_m_), Liquidus (*T*_L_) and dilatometric softening (*T*_d_) temperature and molar volume (*V*_m_). The thermal expansion coefficient (**α**) and density (*ρ*) were seen to decrease. An assessment of the thermal stability of the glasses was made in terms of their processing window (crystallisation onset, *T*_c,ons_ minus glass transition temperature, *T*_g_), and an increase in the processing window was observed with increasing B_2_O_3_ content. Degradation studies of the glasses revealed that the rates decreased with increasing B_2_O_3_ content and a decrease in degradation rates was also observed as the P_2_O_5_ content reduced from 50 to 45 mol%. MG63 osteoblast-like cells cultured in direct contact with the glass samples for 14 days revealed comparative data to the positive control for the cell metabolic activity, proliferation, ALP activity, and morphology for glasses containing up to 5 mol% of B_2_O_3_.

## 1. Introduction

There is a continually growing interest in the use of glasses for varying biomedical applications. Bioactive glasses are a group of surface reactive glasses which can initiate a range of biological responses by releasing ions into the local environment [1]. The first bioactive glass reported was bioglass discovered by Professor Larry Hench in 1969 [2]. Professor Hench and his colleagues investigated a range of silicate-based compositions, and the composition formed consisted of 45% SiO_2_, 24.5% Na_2_O, 24.5% CaO, and 6% P_2_O_5_ (expressed as wt%) also known as 45S5 [3]. An alternate system of glasses for biomedical applications is totally silica free phosphate-based glasses (PBGs). These PBGs have the property of being completely soluble in aqueous medium, and their degradation rate can easily be altered via addition of different modifier oxides. These unique physical and chemical properties of PBGs have attracted huge interest in their use within the field of biomaterials and tissue engineering [4–6]. 

The structure of PBGs is composed of an inorganic phosphate network in which PO_4_^3−^ tetrahedral units are the main building blocks. The tetrahedra can be described in terms of Q^*n*^ terminology, where *n* represents the number of bridging oxygens (BOs) per PO_4_^3−^ tetrahedron. The prevalence of any particular Q species is dependent on the oxygen-to-phosphorous (O/P) ratio in the glass which is determined by the addition of different metal oxides (MeO) [7]. As the ratio MeO/P_2_O_5_ increases from 0 to 3, the phosphate structural groups pass from Q^3^ to Q^0^ [8]. 

Various formulations of PBGs have been studied extensively for their biocompatibility. Different studies showed that the biocompatibility of these glasses was affected strongly by their degradation rate and associated ion release [6]. Therefore, the biocompatibility of PBGs is expected to be affected by the addition of different metal oxides, which are known to alter the chemical durability of the glasses. Ahmed et al. investigated four invert glasses in the system of P_2_O_5_-CaO-MgO-Na_2_O with fixed phosphate and calcium contents at 40 and 25 mol%, and Na_2_O was replaced with 10–30 mol% MgO [9]. They found that the degradation rates for these glasses decreased with increasing MgO and suggested that the degradation profiles and cytocompatibility properties of these glasses were suitable for bone repair applications.

Cell culture studies of the glasses in the system P_2_O_5_-CaO-MgO-Na_2_O-Fe_2_O_3_ using MG63 cells showed that these glasses possessed good cellular response in terms of cell viability, proliferation, and differentiation [10]. Therefore, PBGs can be synthesised with different network modifiers capable of inducing different biological functions with enhanced biocompatibility. 

A wide variety of glass network structures can be expected from the combination of two glass network formers such as P_2_O_5_ and B_2_O_3_ [11, 12]. NMR analysis of borophosphate glasses revealed that the addition of boron increased the chain lengths (Q^2^ species) in the glass structure [13]. Several studies also showed that addition of B_2_O_3_ to the phosphate network improved the chemical durability and thermal properties of the glasses [14–17]. The addition of B_2_O_3_ was also known to improve the thermal stability of these glasses by suppressing their tendency to crystallise [16, 18], which is a common problem associated with the drawing of phosphate glass fibres as they have a tendency towards crystallisation at the working process temperature [19], particularly with Q^0^ and Q^1^ dominated structures. Therefore, it was hypothesised that addition of boron may overcome this problem and make the fibre drawing process easier especially for glasses with phosphate contents lower than 50 mol%.

Recently, it has been shown that some borate glasses can accelerate the formation of a hydroxyapatite layer and bond to bone in a manner which is comparable to 45S5 glasses [13, 20]. Saranti et al. reported that the bioactivity of pure calcium phosphate glasses was favoured by the presence of boron in the glass network due to the ability of boron to change coordination and attach hydroxyl groups at the surface of the glasses [13]. Nielson highlighted that boron played an important role in bone growing than its maintenance [21]. Boron has been speculated to have stimulated hormones, thus mimicking the effects of oestrogen, producing an oestrogen replacement. Currently, oestrogen treatment is still one of the most effective methods of preventing postmenopausal bone loss [21]. Thus, the delivery of boron via the degradation of boron containing degradable PBGs could be of special interest in biomedical applications.

The initial aim of this study was to investigate the effect of B_2_O_3_ addition on the thermal, degradation, and cytocompatibility properties of PBG glasses in the system P_2_O_5_-CaO-MgO-Na_2_O with phosphate contents fixed at 45 and 50 mol%. The final aim was to produce continuous soluble phosphate-based glass fibres (PGF), which could be used as reinforcement for different resorbable polymers (such as Ploylactic acid) to make composites for different biomedical applications. Dissolution studies of the glasses were conducted in phosphate buffer saline (PBS) solution, and the MG63 osteosarcoma cell line was used for the cytocompatibility studies. Furthermore, the effect of B_2_O_3_ addition on the density and molar volume of the glass system was also evaluated. 

## 2. Materials and Methodology

### 2.1. Glass Preparation

Eight different glass compositions were prepared using sodium dihydrogen phosphate (NaH_2_PO_4_), calcium hydrogen phosphate (CaHPO_4_), magnesium hydrogen phosphate trihydrate (MgHPO_4_·3H_2_O), boron oxide (B_2_O_3_), and phosphorous pentoxide (P_2_O_5_) (Sigma Aldrich, UK) as starting materials. The precursors were mixed together and transferred to a 100 mL volume Pt/5% Au crucible (Birmingham Metal Company, UK) which was then placed in a furnace (preheated to 350°C) for half an hour for the removal of H_2_O. The salt mixtures were then melted in a furnace at 1150°C for 1.5 hours, depending on the glass composition as shown in Table 1. Molten glass was poured onto a steel plate, left to cool, and then ground into powder using a pestle and mortar. Having obtained the *T*_g_ of the glass via DSC (see the thermal analysis section), it was then remelted and casted as 9 mm diameter rods by being poured into a graphite mould preheated at different temperatures (as stated in Table 1). The mould was then held at the casting temperature (see Table 1) for an hour and then allowed to cool to room temperature to remove any residual stress.

### 2.2. Thermal Analysis

Glass pieces of the various compositions were ground to fine powder using a pestle and mortar. The glass transition temperature (*T*_g_) of the glasses was determined using differential scanning calorimeter (DSC, TA Instruments Q10, UK). A sample of each glass composition was heated from room temperature to 520°C at a rate of 20°C min^−1^ in flowing argon gas. The *T*_g_ was extrapolated from the onset of change in the endothermic reaction of the heat flow [22]. 

To determine the onset of crystallisation, peak crystallisation, and melting and liquidus temperatures, an alternate DSC instrument (TA Instruments SDT Q600, UK) was used. Samples were heated from room temperature to a value of *T*_g_ + 20°C at a rate of 20°C min^−1^, held there isothermally for 15 min, and then cooled down at a rate of 10°C min^−1^ to 40°C before ramping up again to 1100°C at a rate of 20°C min^−1^ under flowing argon gas. The samples were subjected to the programmed heating cycle to introduce a known thermal history. A blank run was carried out to determine the baseline which was then subtracted from the traces obtained. The *T*_g_ was determined from the second ramping cycle in the same process discussed above. The first deviation of the DSC curve from the baseline above *T*_g_ before crystallisation peak was taken as the onset of crystallisation temperature. The thermal stability of the glasses was measured in terms of the processing window by taking the temperature interval between *T*_g_ and the onset of crystallisation temperature (*T*_c,ons_) as shown in the following:

(1) $$\begin{array}{c} \text{processing window}=T_{\text{c,ons}}-T_{\text{g}} \end{array}$$

### 2.3. Thermomechanical Analysis

The thermal expansion coefficient (*α*) and dilatometric softening temperature (*T*_d_) were measured using a thermomechanical analyser TMA (TA Instruments TMA Q400, UK). Three samples from each composition of an average 7 mm height and 9 mm diameter were heated at a rate of 5°C min^−1^ with an applied load of 50 mN. The measured thermal expansion coefficient (*α*) was taken as an average between 50°C and 250°C. The *T*_d_ was taken as the midway point (on the ordinate) between the abrupt increase in expansion and the onset of contraction.

### 2.4. Helium Pycnometry

The density of the glasses was determined by using a MicromeriticsAccuPyc 1330 helium pycnometer (Norcross, GA, USA). The equipment was calibrated using a standard calibration ball (3.18551 cm^3^) with errors of ±0.05%. Bubble-free bulk glass samples, with an average weight of approximately 6.5 g, were used for the density measurements, and the process was repeated three times. For each composition the molar volume (*V*_m_) was also calculated using the following equation:

(2) $$\begin{array}{c} V_{\text{m}}=\frac{\sum \left(x_{i}\cdot M_{i}\right)}{\rho }, \end{array}$$

where *ρ* is the density, *x*_*i*_ is the molar fraction of *i* component, and *M*_*i*_ is the molecular weight for component *i*. 

### 2.5. Powder X-Ray Diffraction Analysis

The amorphous state of all the glass compositions was confirmed using X-ray diffraction spectra obtained from a Bruker D500 X-ray diffractometer at room temperature with Ni-filtered CuK*α*-radiation (*λ* = 0.15418 nm), operated at 40 kV and 40 mA. The angular range 2*θ* for each scan was from 15° to 100° with a step size of 0.02° and a step time of 0.5 s.

### 2.6. Dissolution Studies

Glass rods of 9 mm diameter were cut into 5 mm thick discs using a low speed saw (South Bay Technologies, CA, USA) equipped with a diamond blade (Buehler, Coventry, UK). Ethanol (Fisher Chemicals, UK) was used as a lubricating fluid. The surface area of the glass discs was measured using a micrometer, and the discs were placed in glass vials containing 30 mL of phosphate buffer solution (PBS). These vials were then placed into an incubator at 37°C. At various time points, the discs were taken out from the vials, excess moisture was removed by blotting the samples dry with tissue, and the area and mass were measured. The dissolution study was carried out for 60 days, and the PBS solution was changed at each time point. During the first week, the mass loss and surface area were measured every day. For the following weeks, the measurements were taken twice a week. Thus the time points were day 1, 2, 3, 4, 7, 10, 14, 16, 20, 23, 27, 30, 34, 36, 44, 47, 50, 54, and 60. The rate of mass loss (%) was calculated according to the following equation:

(3) $$\begin{array}{c} \text{mass loss}\left(\%\right)\;=\;\frac{M_{0}-M_{t}}{M_{0}}, \end{array}$$

where *M*_0_ is the initial mass (g) and *M*_*t*_ is the mass at time *t*. The mass loss per area data was plotted as weight loss per unit area against time. The slope of this graph gave the dissolution rate in terms of g cm^−2^ h^−1^.

### 2.7. Cell Culture

MG63 cells (human osteosarcoma), obtained from the European Collection of Cell Cultures (ECACC), were cultured in complete Dulbecco's Modified Eagle Medium (CDMEM) consisting of DMEM supplemented with 10% foetal calf serum (FCS), 2% HEPES buffer, 2% penicillin/streptomycin, 1% glutamine, 1% nonessential amino acids (Gibco Invitrogen, UK), and 0.85 mM of ascorbic acid (Sigma Aldrich, UK). Cells were cultured in 75 cm^3^ flasks (Falcon, Becton, Dickinson and Company, UK) at 37°C in a humidified atmosphere with 5% CO_2_. The glass discs were sterilised using dry heat at 190°C for 15 minutes and washed three times with sterilised PBS prior to cell culture. Tissue culture plastic (TCP) was used as an internal control for cell growth. Cells were seeded onto the disc sample surfaces at 40,000 cells/cm^2^ concentration and incubated at 37°C in a humidified atmosphere with 5% CO_2_.

### 2.8. Cell Viability/Metabolic Activity

At designated time points, culture medium was removed from the wells, and the samples were washed three times with warm PBS. Alamar Blue solution (1 : 9 Alamar Blue: warm hanks balanced salt solution (HBSS)) (1 mL) was added to each well and incubated for 90 minutes. From each well, 100 *μ*L aliquots were transferred to a 96 well-plate in triplicate, and fluorescence was measured at 530 nm excitation and 590 nm emission using FLx800 microplate reader (BioTek Instruments Inc.).

### 2.9. Proliferation

At designated time points cell culture media was removed, and the samples were washed three times with warm PBS prior to addition of 1 ml deionised water to each well. Cells were lysed using a freeze/thaw technique three times.

100 *μ*L aliquots of cell lysate were transferred to a 96-well plate. DNA standards were prepared using calf thymus DNA (Sigma, UK) and TNE buffer (10 mMTris, 2 M NaCl, and 1 mM EDTA in deionised water, adjusted to pH 7.4) as a diluent. One hundred microlitres of Hoechst stain 33258 was added to each well (1 mg of bis-benzimide 33258 in deionised water, further diluted to 1 : 50 in TNE buffer), and the plate was agitated. Fluorescence was measured at 360 nm excitation and 460 nm emission using a FLx800 microplatefluorimeter (BioTek Instruments Inc.). DNA concentrations were derived from a standard curve of known DNA concentrations generated by the software (KCjunior).

### 2.10. Alkaline Phosphatase Activity

Alkaline phosphatase activity was measured using the Granutest 25 alkaline phosphatase assay (Randox, UK). A 50 *μ*L aliquot of cell lysate (as prepared for DNA quantification assay) was added to a 96-well plate along with 50 mL of the alkaline phosphatase substrate (p-nitrophenyl phosphate in diethanolamine HCI buffer, pH 9.8) and shaken gently, and the absorbance was measured at wavelengths of 405 and 620 nm using a ELx800 microplate colorimeter (BioTek Instruments Inc).

### 2.11. Morphology

Samples were washed with warm PBS at 37°C and fixed in 3% glutaraldehyde in 0.1 M nacacodylate buffer for 30 minutes. After 30 minutes, the fixative was replaced by 7% sucrose solution. Fixed samples were then washed twice in 0.1 M Na-cacodylate buffer and postfixed in 1% osmium tetroxide in PBS for 45 minutes in a fume cupboard. Samples were dehydrated through a graded ethanol series (20, 30, 40, 50, 60, 70, 80, 90, 96, and 100% in water) for approximately 5 minutes each and then dried via hexamethyldisilazane (HMDS) before being sputter-coated in platinum and viewed with a Philips XL30 scanning electron microscope operated at 10 kV.

### 2.12. Statistical Analyses

Average values and standard deviation were computed, and statistical analysis was performed using the Prism software package (version 3.02, GraphPad Software, San Diego, CA, USA, http://www.graphpad.com). Two-way analysis of variance (ANOVA) was calculated with the bonferroni post-test to compare the significance of change in one factor with time. The error bars presented represent standard deviation with *n* = 3.

## 3. Results

### 3.1. Powder X-Ray Diffraction Analysis

**Figure 1:**
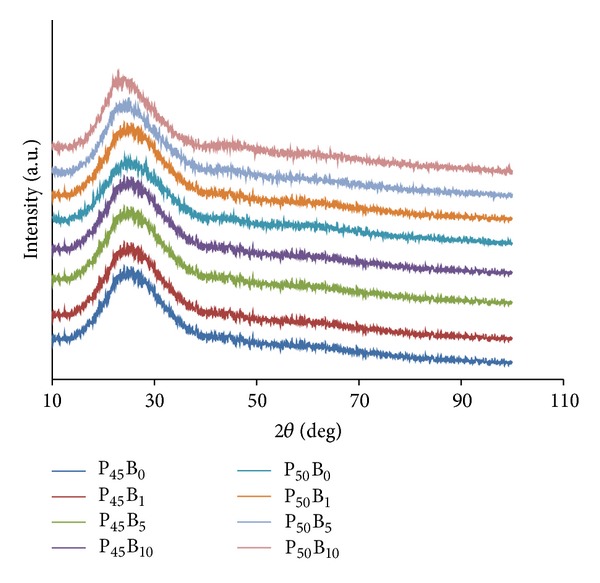
Powder X-ray diffraction pattern for glasses in the system of P_45_Ca_16_Mg_24_Na_(15−*x*)_B_*x*_ and P_50_Ca_16_Mg_24_Na_(10−*x*)_B_*x*_.

XRD traces for the glass samples are presented in Figure 1 below, where a single broad peak between 20° and 40° was observed for each composition. The absence of any sharp crystalline peaks confirmed that all the glasses produced were amorphous.

### 3.2. Thermal Analysis

**Figure 2:**
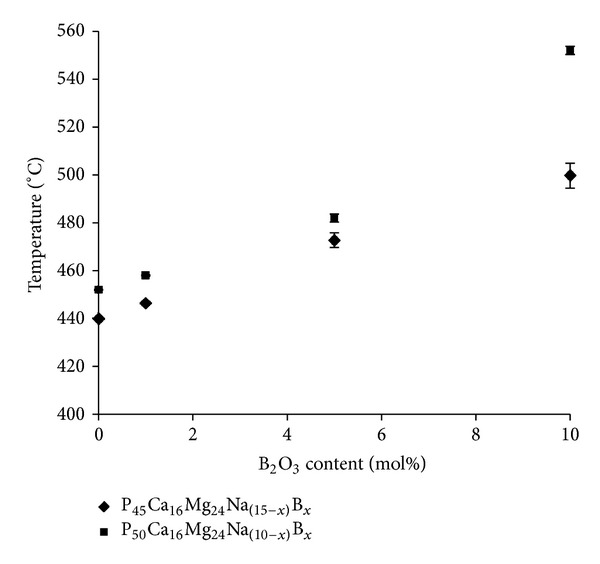
Glass transition temperature (*T*_g_) as a function of the B_2_O_3_ content (mol%) in the system of P_45_Ca_16_Mg_24_Na_15−*x*_B_*x*_ and P_50_Ca_16_Mg_24_Na_(10−*x*)_B_*x*_ (*x* =   0–10) glass system.

**Figure 3:**
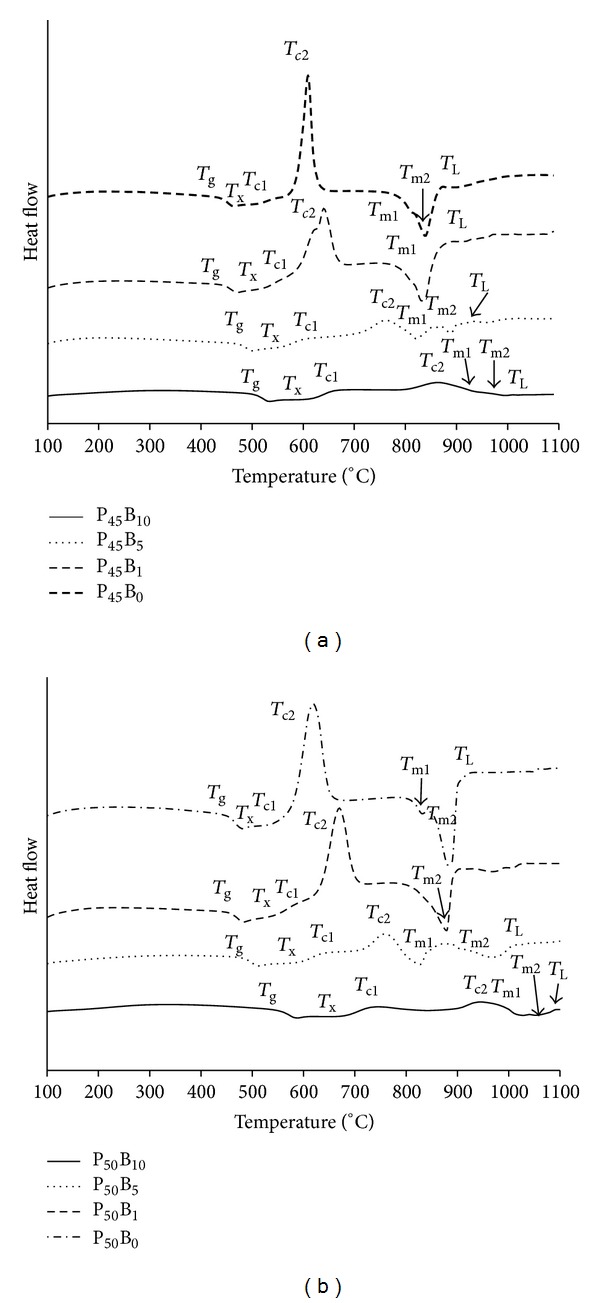
Thermal scans for the glass series in the (a) P_45_Ca_16_Mg_24_Na_(15−*x*)_B_*x*_ and (b) P_50_Ca_16_Mg_24_Na_(10−*x*)_B_*x*_ (*x* = 0–10 mol%) glass system.

Figure 2 shows the effect of increasing B_2_O_3_ content on *T*_g_ values for glasses in the P_45_Ca_16_Mg_24_Na_15−*x*_B_*x*_ and P_50_Ca_16_Mg_24_Na_(10−*x*)_B_*x*_  systems (where *x* = 0,1, 5, or 10). The *T*_g_ values increased with increasing B_2_O_3_ content. An increase in *T*_g_ was also observed with increasing P_2_O_5_ content from 45 to 50 mol%. An increase of 50°C in *T*_g_ values was observed with increasing P_2_O_5_ content from 45 to 50 mol% for the 10 mol% B_2_O_3_ containing glasses, whilst, for the other glass formulations investigated, a difference of *≈*10°C in *T*_g_ values was observed with increasing P_2_O_5_. For glasses containing 45 mol% P_2_O_5_ (P45), the *T*_g_ values increased from 440 to 500°C as Na_2_O was replaced by B_2_O_3._ For glasses with fixed 50 mol% P_2_O_5_ (P50), an increase from 452 to 552°C was observed. Thermal scans for the series of glasses investigated in the P_45_Ca_16_Mg_24_Na_(15−*x*)_B_*x*_ and P_50_Ca_16_Mg_24_Na_(10−*x*)_B_*x*_ systems are shown in Figures 3(a) and 3(b), respectively. The corresponding onset of crystallisation (*T*_c,ons_), crystallisation peak (*T*_c_), melting peak (*T*_m_), and liquidus (*T*_L_) temperature have been labelled on the thermal traces obtained.

The onset of crystallisation was found to increase with increasing boron content in each glass system. The thermal scans of all the compositions exhibited two crystallisation (*T*_c1_ and *T*_c2_) and melting peaks (*T*_m1_ and *T*_m2_); in addition, the intensity of the crystallisation peaks decreased and became less defined with increasing boron content. A summary of *T*_c,ons_, *T*_c_, *T*_m_, and *T*_L_ is given in Table 2. 

**Figure 4:**
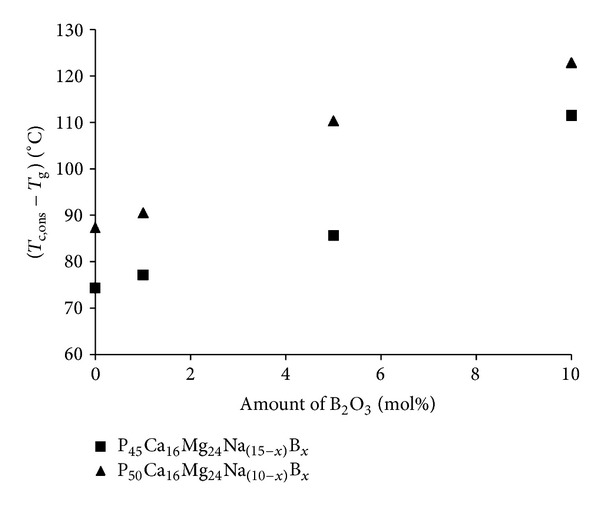
Processing window (crystallisation onset, *T*_c,ons_ minus glass transition temperature, *T*_g_) as a function of B_2_O_3_ content (0–10 mol%).

The values for the processing window (*T*_c,ons_ − *T*_g_), which is also an indication of the thermal stability for glasses, are presented in Figure 4. The values for the processing window were seen to increase with increasing B_2_O_3_ content for both the P45 and P50 glasses. With 5 mol% B_2_O_3_ addition, the processing window increased from 86°C to 110°C with increasing P_2_O_5_ content from 45 to 50 mol%. The processing window for P45B0 and P50B0 glasses was 74°C and 87°C, respectively. Whereas, the processing window increased to 112°C and 123°C for P45B10 and P50B10 glasses, respectively.

### 3.3. Thermal Expansion Coefficient and Dilatometric Softening Temperature Measurements

**Figure 5:**
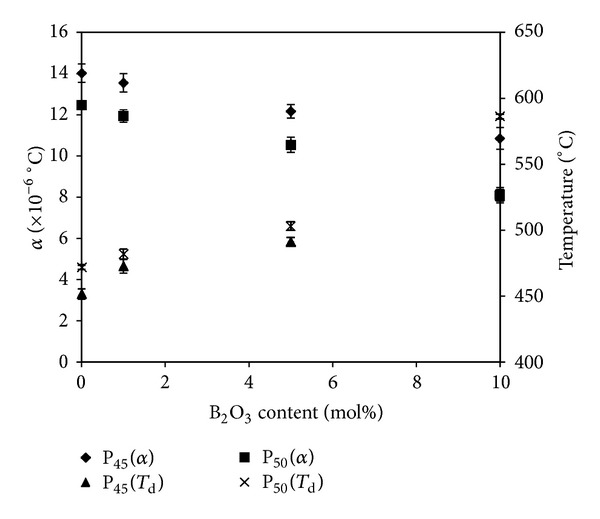
Thermal expansion coefficient (*α*) and dilatometric softening temperature (*T*_d_) of the glasses in the system of P_45_Ca_16_Mg_24_Na_(15−*x*)_B_*x*_ and P_50_Ca_16_Mg_24_Na_(10−*x*)_B_*x*_(*x* = 0–10) as a function of B_2_O_3_ content (mol%). The values of *α* is measured in the range between 50 and 250°C.

Dilatometric measurements provided the values for thermal expansion coefficient *α* (measured between 50°C and 250°C) and the dilatometric softening temperature (*T*_d_) (see Figure 5). The values for *α* were seen to decrease with increasing B_2_O_3_ content from 14 × 10^−6°^C to 11 × 10^−6°^C and from 12 × 10^−6°^C to 8 × 10^−6°^C for P45 and P50 glasses, respectively. The dilatometric softening temperatures increased from 452°C to 526°C and from 472°C to 586°C with 10 mol% B_2_O_3_ addition to the P45 and P50 glasses, respectively.

### 3.4. Density and Molar Volume Measurements

**Figure 6:**
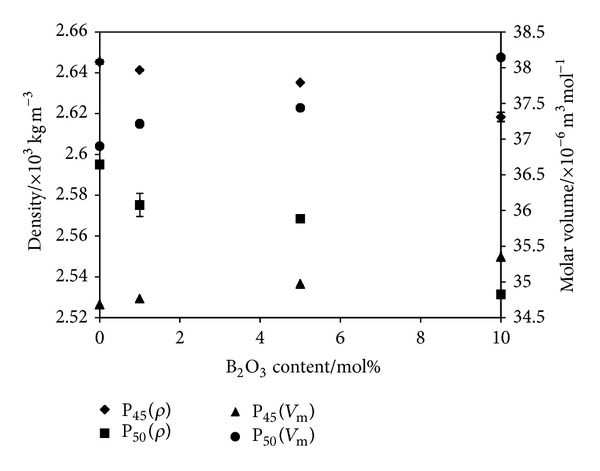
Plot of density (*ρ*) and molar volume (*V*_m_) of the glasses in the system of P_45_Ca_16_Mg_24_Na_(15−*x*)_B_*x*_ and P_50_Ca_16_Mg_24_Na_(10−*x*)_B_*x*_ (*x* = 0–10) as a function of B_2_O_3_ content (0–10 mol%).

Figure 6 shows the variation in density (*ρ*) and molar volume (*V*_m_) with increasing B_2_O_3_ content of the glasses. The density was seen to decrease from 2.65 to 2.62 × 10^3^ kg m^−3^ for P45 glasses and from 2.59 to 2.53 × 10^3^ kg m^−3^ for P50 glasses with increasing boron content. Consequently, for the P45 glasses the molar volume increased from 34.7 to 35.4 × 10^−6^ m^3^ mol^−1^, and for the P50 glasses an increase from 37.0 to 38.14 × 10^−6^ m^3^ mol^−1^ was observed with increasing boron content.

### 3.5. Glass Solubility

**Figure 7:**
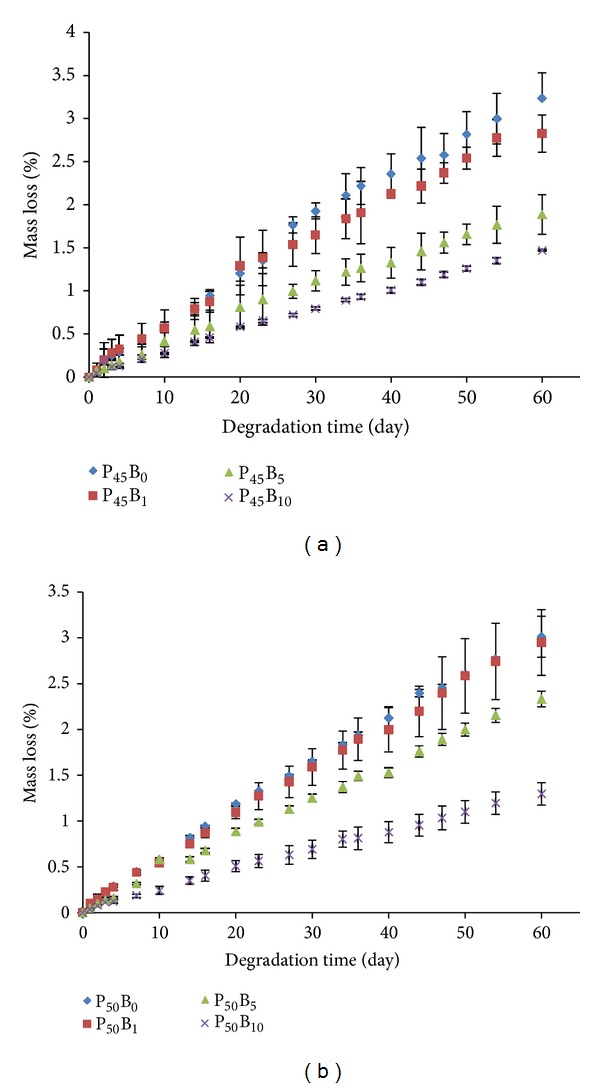
Plot of mass loss (%) of the glasses in the system of (a) P_45_Ca_16_Mg_24_Na_(15−*x*)_B_*x*_ and (b) P_50_Ca_16_Mg_24_Na_(10−*x*)_B_*x*_ (*x* = 0–10) in PBS at 37°C for 60 days.

Figures 7(a) and 7(b) show the mass loss (%) of the glasses as a function of immersion time in phosphate buffer solution at 37°C for P45 and P50 glasses, respectively. No significant difference (*P* < 0.05) in mass loss was seen for glasses containing 0 and 1 mol% B_2_O_3._ However, with further increase in B_2_O_3_ content (5–10 mol%), the mass loss decreased. At 10% B_2_O_3_ addition, the total mass loss observed over the full duration of the 60-day study was seen to decrease from 3.01 to 1.30% for P45 glasses, and for P50 glasses a reduction from 3.24 to 1.40% was observed.

**Figure 8:**
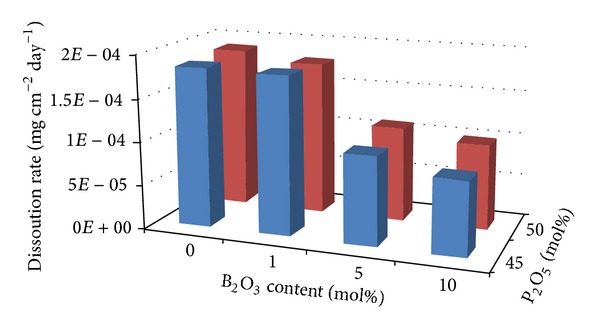
3D graph of dissolution rate values obtained for glasses fixed with 45 and 50 mol%.

Figure 8 is a 3D representation of the dissolution rates obtained for the glasses investigated. With addition of B_2_O_3_ at the expense of Na_2_O, the dissolution rate decreased. After 60 days, the dissolution rate for the P45B0 and P50B0 glasses was 1.85 × 10^−4^ and 1.89 × 10^−4 ^g cm^−1^ day^−1^, whilst the dissolution rate decreased to 8.57 × 10^−5^ and 9.85 × 10^−5 ^g cm^−1^ day^−1^ for P45B10 and P50B10 glasses, respectively. A decrease in dissolution rate was also observed with decreasing P_2_O_5_ content from 50 to 45 mol%. 

### 3.6. Cytocompatibility Studies

#### 3.6.1. Cell Viability/Metabolic Activity

**Figure 9:**
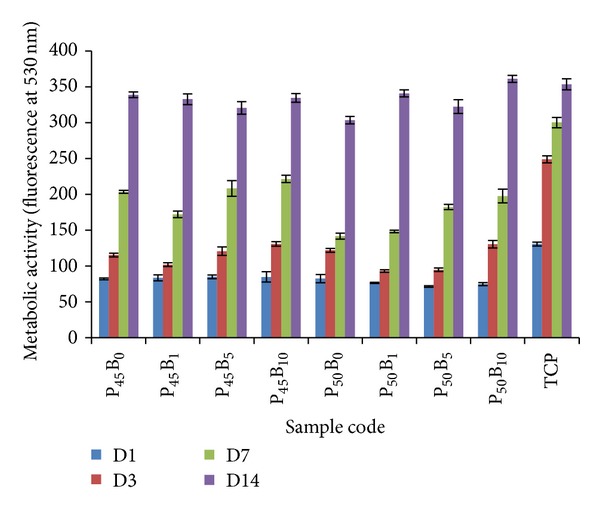
Metabolic activity of MG63 cells, as measured by the Alamar Blue assay, cultured on PBGs. The time points are day 1, 3, 7, and 14. Error bars represent the standard deviation where *n* = 6.

The Alamar Blue assay was used to determine the effect of boron addition on the metabolic activity of osteoblast-like cells (MG63). The cells were cultured for 14 days, and the time points were day 1, 3, 7, and 14 (Figure 9).

Metabolic activity was seen to increase throughout the 14-day culture period. The TCP control demonstrated faster metabolic activity compared to other samples at early time points (up to 7 days), which was statistically significant (*P* < 0.001). Cell metabolic activity on P50 glasses, especially P50B0 and P50B1, was seen to be significantly lower (*P* < 0.01) on the 7th day compared to the P45 glasses. However, at the latter time points all glass samples demonstrated similar levels of metabolic activity to the TCP control. 

#### 3.6.2. Alkaline Phosphatase Activity

**Figure 10:**
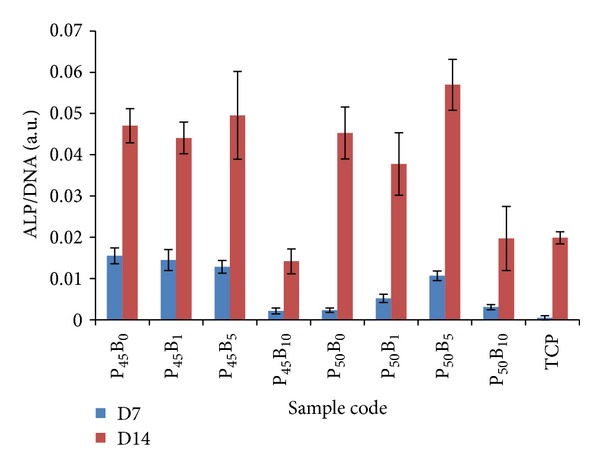
Alkaline phosphate activity of MG63 cultured on PBGs. The data was normalised to DNA concentration. The time points are day 1, 3, 7, and 14. Error bars represent the standard deviation where *n* = 6.

The effect of boron addition to PBGs on the osteoblast-like phenotype was analysed by measuring the alkaline phosphatase activity of osteosarcoma cells (see Figure 10). Data was normalised with the corresponding DNA concentration at each time point.

For all samples, including the TCP (internal control), the ALP activity was not detectable up to 3 days. However, after 7 days of culture, detectable amounts of ALP activity were observed on all samples, with notably overall higher ALP activity on P45 formulations. The ALP activity of cells cultured on P45 PBG containing 0–10% boron was significantly higher (*P* < 0.05) than the other glass samples investigated with the exception of glass code P50B5. After 14 days, no significant difference (*P* > 0.05) was observed between P45 and P50 containing 0–5% boron. However, both P45 and P50 glasses containing 10% boron appeared to have a less influence on ALP compared to 5 mol% B_2_O_3_ doped glasses; nonetheless, they were still comparable to the TCP control (*P* > 0.05).

#### 3.6.3. Morphology

**Figure 11:**
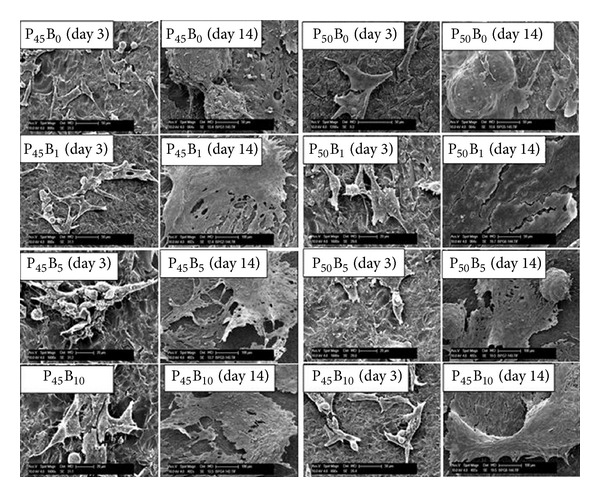
SEM images of MG63 cells cultured on P_45_Ca_16_Mg_24_Na_(15−*x*)_B_*x*_ (left) and P_50_Ca_16_Mg_24_Na_(10−*x*)_B_*x*_ (right) after 3 days and 14 days of culture. Micrometer scale bar = 50 *μ*m.

The morphology of the cells cultured on phosphate glass discs was visualised using SEM (see Figure 11). A representative image of MG63 osteosarcoma cells cultured on phosphate glass specimens for 14 days is presented. In general, cells cultured on all glass surfaces showed a confluent layer after 14 days of culture. However, glass code P50B0 (i.e., containing no boron) appeared to have promoted formation of large nodules of cells which resulted in a nonconfluent layer after 14 days of culture. At initial time points (1, 3 days) glasses containing 1 and 5 mol% boron had greater cell density and attachment sites (lamellipodia and filopodia) compared to glass containing 10 mol% boron oxide. Due to the inherent (cancerous) nature of MG63 cells, large nodules of cells were found at initial time points which resulted in the formation of a dense cell layer at later time points. This observation was consistent for all the glass samples. However, no clusters were spotted at any time points on the TCP control, where the cells were densely packed showing spindle-shaped cells.

## 4. Discussion

In the absence of any modifier oxides, the structure of PBGs consists of PO_4_ groups with many non-bridging oxygens, which explains the low melting temperatures and poor chemical durability of PBGs [7]. For PBGs to be applicable in biomedical applications, the composition of the glasses can be tailored to have suitable dissolution properties. Addition of B_2_O_3_ to sodium aluminium phosphate glasses was found to increase their *T*_g_ with almost complete elimination of crystallisation [23]. Similar results with boron addition were also reported for different phosphate glass systems, and their improvement in thermal properties was ascribed to the formation of BO_4_ structural units within the phosphate network [16]. 2 mol% boron-modified 45S5 bioglass was found to promote new bone formation more rapidly than standard 45S5 bioglass particles upon implantation into tibial defects in rats [24]. Liang et al. found that scaffolds made with sodium calcium borate glass were converted into hydroxyapatite within 6 days of immersion into K_2_PO_4_, which supported the attachment and differentiation of human bone marrow derived mesenchymal stem cells and human mesenchymal stem cell derived osteoblasts [25].

All the samples investigated in this study were shown to be amorphous (see Figure 1). It is well known that the physical and thermal properties of glasses are strongly dependent on their structure and composition [11, 15]. As seen from the DSC results, the *T*_g_ values increased progressively with the replacement of Na_2_O with B_2_O_3_. Increases of 60°C and 102°C were observed, respectively, for P45 and P50 glasses with 10 mol% B_2_O_3_ additions, as compared to glasses without B_2_O_3_. This increase was attributed to the fact that the B-O bond strength is four times higher than that of Na-O bond strength as B^3+^ has a much smaller ionic radius (0.41 Å) compared to Na^+^ (1.13 Å). A similar effect on *T*_g_ values for replacing Na^+^ with Ti^4+^ was observed by Abou Neel et al. where *T*_g_ values increased by 155°C with 15 mol% TiO_2_ addition due to the smaller ionic radius of Ti^4+^ (0.56 Å) compared to Na^+^ [26]. Moreover, Higby et al. reported that replacement of Na_2_O with B_2_O_3_ caused a decrease in the number of non-bridging oxygens which is also known to increase the *T*_g_ [27]. The *T*_g_ values were also found to increase as the P_2_O_5_ content increased from 45 to 50 mol%. Several studies have shown that glasses with 50 mol% P_2_O_5_ content are dominant in long chains or rings (i.e., Q^2^ species), whilst both Q^1^ and Q^2^ species have been identified for glasses with 45 mol% P_2_O_5_ [4, 28]. Thus, reducing the P_2_O_5_ content from 50 to 45 mol% depolymerises the glass network via the introduction of shorter chain Q^1^ species which would account for the higher *T*_g_ values for P50 glasses as compared to P45 formulations. Ahmed et al. also showed a similar decreasing trend in *T*_g_ values with decreasing P_2_O_5_ content from 50 to 45 mol%; however, with increasing P_2_O_5_ content from 50 to 55 mol%, the *T*_g_ value decreased [28].

An assessment of the thermal stability of the glasses was made in terms of the processing window. Crystallisation temperatures increased with increasing B_2_O_3_ addition, and the processing window increased (Figure 4). Massera et al. studied the thermal properties of glasses in the system SiO_2_-Na_2_O-B_2_O_3_-CaO-MgO-Al_2_O_3_-P_2_O_5_ and found that the onset of crystallisation temperature increased as Na_2_O was replaced with B_2_O_3_ [15]. They attributed this change to a reduction in non-bridging oxygens with decreasing amounts of Na_2_O [15]. Harada et al. studied glasses in the system BaO-P_2_O_5_-B_2_O_3_ and reported that the addition of B_2_O_3_ suppressed the formation of orthophosphate Q^0^ units, which promotes crystallisation. They also suggested that the addition of B_2_O_3_ suppressed surface crystallisation due to the formation of borate anions and the cross-linked chain structure based on metaphosphate Q^2^ tetrahedra [16]. Pemberton et al. and Saranti et al. also suggested that crystallisation occurred from the short chain regions of the glass and that addition of boron could alter the dimensionality of the phosphate network via the formation of long chain Q^2^ species rather than Q^0^ or Q^1^ units [13, 29]. Thus, addition of boron to the phosphate glass network could increase the cross-linking density and also increase the chain length, which in turn would improve the processing window of the glass system as seen from Figure 4.

Initial trials to draw these glass formulations into fibres provided empirical evidence that the larger processing windows obtained due to B_2_O_3_ incorporation facilitated ease of fibre fabrication for these formulations, as compared to the nonboron containing controls. Continuous fibres of 18–20 micron diameter were successfully drawn from the glass formulation (P_2_O_5_)_45_-(CaO)_16_-(Na_2_O)_10_-(MgO)_24_-(B_2_O_3_)_5_ with tensile strength and modulus of 1050 ± 165 MPa and 59.6 ± 5 GPa, respectively. 

In addition, through TMA analysis, it was observed that with increasing B_2_O_3_ content the thermal expansion coefficient decreased, whereas the *T*_d_ temperature increased (Figure 5). The thermal expansion coefficient of PBGs is strongly dependent on the cross-link density of the glass network and also on the interaction of the cations with the non-bridging oxygens in the phosphate chain [30]. The cationic field strength is a measure of a cation's effective force for attracting anions and is given by *Z*/*r*, where *Z* is the valency and *r* is the ionic radius of the cation [31]. Therefore, as B^3+^ has a smaller ionic radius than Na^+^ (as discussed above), and the valency is also higher, the field strength of B^3+^  would therefore also be higher than Na^+^. This higher field strength of B^3+^  would have a stronger ability to undergo coordination with other groups. The Raman and infrared spectra of the glasses containing B_2_O_3_ have showed that the addition of B_2_O_3_ to the phosphate glass structure can form highly cross-linked BPO_4_ units which are composed of interconnected BO_4_ and PO_4_ tetrahedral units [32, 33]. Thus, the higher field strength of B^3+^ compared to Na^+^ along with an increase in cross-link density with increasing B_2_O_3_ content resulted in an increase in *T*_d_ and decreased the vibrational movement of the basic structural units, resulting in a decrease in the thermal expansion coefficient values.

The density of the glasses was found to decrease with increasing B_2_O_3_ content (Figure 6), whereas the molar volume increased (Figure 6). Qiu et al. investigated the density of glasses in the ternary system P_2_O_5_-B_2_O_3_-Na_2_O and also found that replacement of Na_2_O with B_2_O_3_ decreased the density of their glasses [34]. It was suggested that the density of Na_2_O was higher than B_2_O_3_ in the bulk which could have caused the decrease in density observed with B_2_O_3_ addition. They also reported that, as B_2_O_3_ is a network former, the density values may not always reflect the packing density within the glass structure. EL-Hadi et al. studied the density, and molar volume of, a series of sodium borosilicate glasses and found that, by replacing B_2_O_3_ with Na_2_O, the density of the glasses increased, and molar volume decreased [35]. They suggested that addition of Na_2_O to the borosilicate glasses increased the amount of non-bridging oxygens and thus reduced the volume which in turn increased the density and decreased molar volume. In this study, a reduction in non-bridging oxygens was expected as Na_2_O was replaced with B_2_O_3_, which correlated well with the existing literature. 

A decrease of 55% and 57% in mass loss was observed at 10 mol% B_2_O_3_ containing P45 and P50 glasses over the time period of 60 days as compared to the glasses with no boron (Figure 7). This decrease in mass loss (%) and dissolution rate with increasing B_2_O_3_ was attributed to the replacement of P–O–P bonds with P–O–B bonds, which correlated well with an increase in *T*_g_ and decrease in thermal expansion coefficient. The chemical bond strength of the diatomic molecule of B–O (808 kJ/mol) is higher than that of P–O (599.1 kJ/mol), suggesting an energetic preference for increased bonding forces inside the glass structure via formation of P–O–B bonds [36]. At higher B_2_O_3_ content the BO_4_ structural unit increased replacing P–O–P bonds with P–O–B bonds which gradually reduced the degradation rate. Shah et al. studied the degradation behaviour of phosphate glasses in the Na_2_O-BaO-B_2_O_3_-P_2_O_5_ quaternary system and found that the dissolution rate of the glasses decreased with increasing B_2_O_3_ content [37] which also correlated well with this study. 

However, the degradation rate of the glasses was not only affected by the increasing addition of B_2_O_3_, but the amount of P_2_O_5_ also affected this dissolution rate. Brauer et al. found that the degradation rate of PBGs reduced by two orders of magnitude as the P_2_O_5_ content was reduced from 50 to 45 mol%. Vogel et al. suggested that the reduced degradation rate was due to depolymerisation of the phosphate chain structure from Q^2^ to Q^1^ species as the Q^2^ structures were more susceptible to hydration and subsequent hydrolysis as compared to Q^1^ structures [38]. However, it has been reported that the hydration energy for the phosphates decreased in the order of Q^2^ > Q^1^ > Q^0^ [39].

The cytocompatibility results showed that incorporation of B_2_O_3_ (up to 10 mol%) into the glass systems investigated showed no detrimental effect on cell metabolic activity which was comparable to the TCP control (see Figure 9). Cell morphology was also shown to be unaffected for glasses containing B_2_O_3_ (Figure 11). However, the metabolic activity was significantly lower (*P* < 0.001) for the P50 formulations as compared to the P45 formulations, especially at the initial time points (up to day 7). Hasan et al. [10] reported that the cell metabolic activity and ALP activity of quinternary formulations containing 40–45% P_2_O_5_ were better than glasses fixed with 50 mol% P_2_O_5_. They suggested that increased amounts of inorganic phosphate ions released from glass samples containing 50 mol% P_2_O_5_ could be responsible for the lower cell metabolic and ALP activities as excessive amounts of inorganic phosphate ions are known to be detrimental for cell functions [40]. Ma et al. [41] also reported that the release of excessive inorganic phosphate ions from hydroxyl apatite in cell culture media reduced osteoblast differentiation and mineralisation. 

In the current study, ALP activity on all glass samples containing 0–5% B_2_O_3_ was greater than the TCP control (see Figure 10). A very recent study was carried out to observe the effect of boron (0, 1, 10, 100, and 1000 ng/mL) on osteogenic differentiation of human bone marrow stromal cells (BMSCs), and the results indicated that BMSCs treated with 10 and 100 ng/mL boron presented a higher ALP activity compared to the control [42]. Abou Neel et al. reported that the ALP activity of the phosphate glasses in the system of P_2_O_5_-CaO-Na_2_O was reproducibly enhanced as 3 and 5 mol% TiO_2_ were added to the glass system. The authors suggested that this enhancement may be associated with the lower degradation of these compositions which help to maintain the pH at a level favourable for the cellular activity [43]. Ahmed et al. investigated the effect of Fe_2_O_3_ addition on the CaO-Na_2_O-P_2_O_5_ ternary glass system and found a positive effect of increasing Fe_2_O_3_ content on cell attachment and differentiation and attributed this towards the increased durability of the glass system [28]. Thus, it could be hypothesised that the improved durability of the glasses with B_2_O_3_ addition was responsible for the higher ALP activity for the glasses investigated. However, although comparable with the TCP control, the ALP activity of the glasses with 10 mol% B_2_O_3_ was significantly lower than other glass samples (*P* > 0.01). The lower ALP activity observed for cells cultured on glass samples containing 10% B_2_O_3_ could possibly be associated with boron ions acting as a stabilising agent for the alkaline phosphatase enzyme [44], which could have cross-linked the enzyme causing lower cleavage of paranitrophenol phosphate (colourless) to paranitriphenol + phosphate (yellow). Zhang et al. [45] investigated porous scaffolds of bioactive borosilicate glasses in the system R_2_O-RO-B_2_O_3_-SiO_2_-P_2_O_5_ with four different contents of borate. It was reported that high cumulative concentrations of boron ions had an inhibitory effect on cell proliferation. 

The studies here have shown that boron addition to PBGs has a significant effect on the glass properties via extension of the phosphate chain length and increased processing window. This suggests that continuous manufacture of fibres from glasses with lower phosphate containing content would be possible. This will be investigated in the follow-up study. 

## 5. Conclusions

Eight different phosphate based glass compositions in the system P_2_O_5_-CaO-Na_2_O-MgO-B_2_O_3_ were produced by replacing the Na_2_O with B_2_O_3_, whilst the P_2_O_5_ content was fixed to 45 and 50 mol%. The *T*_g_ and *T*_d_ temperatures increased as Na_2_O was replaced with B_2_O_3_. For both P45 and P50 glasses, the highest *T*_g_ was for glasses with 10 mol% B_2_O_3_. The thermal stability of the glasses was assessed in terms of a processing window and compared to glasses with no boron; the processing window for P45 and P50 glasses increased by 38°C and 36°C with 10 mol% addition of B_2_O_3_. The thermal expansion coefficient values, density, and dissolution rate decreased with increasing B_2_O_3_, whereas the molar volume increased. The lowest thermal expansion coefficient (8 × 10^−6°^C) and density (2.50 × 10^3^ kg m^−3^) were observed for glass P50B10. The degradation study revealed a decrease of 55% and 30% in degradation rate for glass codes P45B10 and P50B10 as compared to the controls (P45BO and P50B10). Incorporation of boron into the glass systems investigated showed favourable effects on the cell metabolic activity, proliferation, and morphology. The ALP activity improved for glasses containing 0–5% B_2_O_3_.However, higher (10%) boron content appeared to have no influence on ALP activity when compared with the TCP control.

## Figures and Tables

**Table 1:** Glass codes and drying, melting, and casting temperatures used throughout the study.

Glass code	P_2_O_5_ content (mol%)	CaO content (mol%)	Na_2_O content (mol%)	MgO content (mol%)	B_2_O_3_ content (mol%)	Drying temp/time (°C/h)	Melting temp/time (°C/h)	Casting temp/time (°C/h)
P45B0	45	16	15	24	—	350/1	1150/1.5	450/1
P45B1	45	16	14	24	1	350/1	1150/1.5	456/1
P45B5	45	16	10	24	5	350/1	1150/1.5	484/1
P45B10	45	16	5	24	10	350/1	1150/1.5	510/1
P50B0	50	16	10	24	—	350/1	1150/1.5	460/1
P50B1	50	16	9	24	1	350/1	1150/1.5	474/1
P50B5	50	16	5	24	5	350/1	1150/1.5	492/1
P50B10	50	16	—	24	10	350/1	1150/1.5	560/1

**Table 2:** The thermal characteristics (*T*_x_, *T*_c_, *T*_m_, *T*_L_) for P_45_Ca_16_Mg_24_Na_(15−*x*)_B_*x*_ and P_50_Ca_16_Mg_24_Na_(10−*x*)_B_*x*_ (*x* = 0–10) glass systems.

Glass code	*T*_c,ons_/°C	*T*_c_/°C	*T*_m_/°C	*T*_L_/°C
P45B0	512.2 ± 0.5	535.4 ± 0.7	809.7 ± 0.1	864.3 ± 3.0
		609.3 ± 0.4	838.0 ± 0.3	
P45B1	513.2 ± 1.3	539.3 ± 1.5	812.3 ± 0.1	864.1 ± 0.1
		609.4 ± 0.3	838.3 ± 0.3	
P45B5	561.6 ± 1.7	559.7 ± 0.8	821.9 ± 0.1	972.0 ± 0.3
		759.8 ± 0.8	892.4 ± 0.3	
P45B10	615.2 ± 3.5	660.1 ± 0.3	932.7 ± 0.3	1011.4 ± 0.4
		865.1 ± 1.0	995.1 ± 1.0	
P50B0	538.2 ± 1.7	614.31 ± 1.7	832.0 ± 0.1	903.10 ± 0.3
			887.4 ± 0.3	
P50B1	543.0 ± 0.3	573.3 ± 0.3	827.9 ± 2.5	901.5 ± 1.0
		654.8 ± 1.0	880.6 ± 0.0	
P50B5	589.9 ± 0.7	637.2 ± 1.0	829.1 ± 0.0	1031.5 ± 0.6
		758.54 ± 4.8	970.51 ± 6.6	
P50B10	669.1 ± 1.5	745.8 ± 0.8	1029.4 ± 0.3	1091.9 ± 0.6
		942.8 ± 0.8	1059.7 ± 0.4	
